# Microbial Diversity in Sulfate-Reducing Marine Sediment Enrichment Cultures Associated with Anaerobic Biotransformation of Coastal Stockpiled Phosphogypsum (Sfax, Tunisia)

**DOI:** 10.3389/fmicb.2017.01583

**Published:** 2017-08-21

**Authors:** Hana Zouch, Fatma Karray, Fabrice Armougom, Sandrine Chifflet, Agnès Hirschler-Réa, Hanen Kharrat, Lotfi Kamoun, Wajdi Ben Hania, Bernard Ollivier, Sami Sayadi, Marianne Quéméneur

**Affiliations:** ^1^Laboratory of Environmental Bioprocesses, LMI COSYS-Med, Biotechnology Center of Sfax Sfax, Tunisia; ^2^Aix Marseille Université, CNRS/INSU, Université de Toulon, IRD, Mediterranean Institute of Oceanography UM 110 Marseille, France; ^3^Department of Research, Tunisian Chemical Group Sfax, Tunisia

**Keywords:** phosphogypsum, marine sediment, anaerobes, sulfate-reducing bacteria, *Desulfovibrio*, next-generation sequencing, anaerobic biotechnology, bioremediation

## Abstract

Anaerobic biotechnology using sulfate-reducing bacteria (SRB) is a promising alternative for reducing long-term stockpiling of phosphogypsum (PG), an acidic (pH ~3) by-product of the phosphate fertilizer industries containing high amounts of sulfate. The main objective of this study was to evaluate, for the first time, the diversity and ability of anaerobic marine microorganisms to convert sulfate from PG into sulfide, in order to look for marine SRB of biotechnological interest. A series of sulfate-reducing enrichment cultures were performed using different electron donors (i.e., acetate, formate, or lactate) and sulfate sources (i.e., sodium sulfate or PG) as electron acceptors. Significant sulfide production was observed from enrichment cultures inoculated with marine sediments, collected near the effluent discharge point of a Tunisian fertilizer industry (Sfax, Tunisia). Sulfate sources impacted sulfide production rates from marine sediments as well as the diversity of SRB species belonging to *Deltaproteobacteria*. When PG was used as sulfate source, *Desulfovibrio* species dominated microbial communities of marine sediments, while *Desulfobacter* species were mainly detected using sodium sulfate. Sulfide production was also affected depending on the electron donor used, with the highest production obtained using formate. In contrast, low sulfide production (acetate-containing cultures) was associated with an increase in the population of *Firmicutes*. These results suggested that marine *Desulfovibrio* species, to be further isolated, are potential candidates for bioremediation of PG by immobilizing metals and metalloids thanks to sulfide production by these SRB.

## Introduction

Phosphogypsum (PG) is the main by-product of the production of phosphate fertilizers. It is produced by the economic wet process, which generates a large amount of PG (Tayibi et al., 2009; Gennari et al., 2011). Worldwide PG production is estimated to be around 100–280 million tons per year (Mt/year; Tayibi et al., 2009), but only a small part is reprocessed (15%), and the majority is stockpiled. In Tunisia, one of the main phosphate producing countries in the world, the phosphate fertilizer plants are mainly located around the Gulf of Gabes and produce 1–10 Mt/year PG (Ajam et al., 2009). In Gabes city, the PG produced is directly discharged into the seawater. In the Sfax and Skhrira plants, the PG is stockpiled for long-term storage without further treatment.

PG consists mainly of calcium sulfate dihydrate (CaSO_4_·2H_2_O), and is characterized by strongly acidic pH (pH ~3) due to the presence of phosphoric acid and sulfuric acid residues (Zairi and Rouis, 1999; Azabou et al., 2005; Zmemla et al., 2016). Depending on the origin of the phosphate ores, PG may contain some trace metals [e.g., Sr (205–1,118 mg/kg), Zn (4–107 mg/kg), Cr (1–75 mg/kg)], as well as radionuclides [e.g., ^226^Ra (15–1,700 Bq/kg); (Ben Amor and Jomaa, 2012)]. These toxic elements prevent the effective reuse of PG in various industrial or agricultural activities (Papastefanou et al., 2006; Tayibi et al., 2009). Moreover, the discharge of PG into the seawater or long-term PG stockpiling in close proximity to the sea present a potential threat to the surrounding coastal and marine ecosystems with substantial impact on human and environmental health and activities.

In order to reduce the amount of stockpiled PG and related trace metals, different biotechnological methods have been recently proposed (Wolicka, 2008; Jalali et al., 2016; Martins et al., 2016). Among them, the anaerobic bio-treatment of PG using pure or mixed cultures of sulfate-reducing bacteria (SRB) grown on simple or complex substrates, such as organic industrial and agricultural wastes has been reported (Azabou et al., 2005; Rzeczycka and Blaszczyk, 2005; Wolicka, 2008; Martins et al., 2016). During the course of these processes, SRB were shown to be particularly active in sulfate reduction from PG into hydrogen sulfide (H_2_S; Azabou et al., 2005). Thereafter, H_2_S may be oxidized into sulfuric acid by sulfide-oxidizing bacteria to be possibly reused in the fertilizer industry to produce phosphoric acid (Pokorna and Zabranska, 2015). The performance of PG biotransformation was shown to depend on bacterial species, inoculum size, PG loading, pH, and temperature, but also type of substrates (Azabou et al., 2005; Rzeczycka and Blaszczyk, 2005). Various types of inocula, such as soil or sludge, have been tested over the last 10 years (Thabet et al., 2016), but it is only recently that one study has explored microbial diversity in such bioprocess (Martins et al., 2016). The ability of PG microbial communities to use sulfate from acidic PG has recently been studied from Portuguese PG (Martins et al., 2016). The SRB cultivated from this PG were mainly related to the *Desulfosporosinus* genus. However, to the best of our knowledge, the composition of total indigenous microbial populations inhabiting Tunisian PG has not yet been reported.

In coastal marine environments, where sulfate is present in large quantities, SRB contribute up to 50% of organic matter mineralization (Jørgensen, 1982). Marine SRB form a diverse and heterogeneous group of microorganisms and belong mainly to the phylum of the *Deltaproteobacteria* (e.g., *Desulfovibrio* spp.) and *Firmicutes* (e.g., *Desulfotomaculum* spp.). Several novel SRB have been isolated from Tunisian marine and coastal polluted sediments near Sfax or Skhrira plants (e.g., *Desulfovibrio marinus, Desulfobulbus aggregans*; Thabet et al., 2007; Kharrat et al., 2017). These bacteria may produce sulfide from sulfate, using a wide range of substrates including hydrogen, organic acids, or alcohols, thus making them good candidates for PG biotransformation from organic wastes. Moreover, H_2_S produced by SRB may react with different trace metals present in PG (e.g., Zn or Cd), to form highly insoluble metal sulfides (Rzeczycka et al., 2004; Azabou et al., 2007a). For instance, Azabou et al. (2007a) showed that Zn was removed by SRB to < 5% from medium containing initially 150 mg/L of Zn. In this respect, biotechnological process based on SRB activity may be considered as environmentally friendly and low-cost alternative for the treatment of metal-rich PG waste. Although, several studies have investigated the ability of SRB to reduce PG mass (more than 2.5 g of PG per liter of medium; Wolicka and Kowalski, 2006; Wolicka and Borkowski, 2009), none of these studies analyzed the fate of trace metals originated from PG in these cultures. Thus, despite some advances in research on PG biotransformation by SRB (bioremediation process), its commercial development has been substantially limited by certain major challenges. These include the efficient reduction or immobilization of co-contaminants (i.e., trace metals, radionuclides), the use of low-cost and abundant renewable water sources for sulfate reduction (e.g., wastewaters and/or seawater), and the improvement of sulfate reduction yields through the development of optimal control of mixed SRB cultures. Despite the abundance of SRB in marine ecosystems and their efficiencies in PG biotransformation, the biotechnological potential, and diversity of marine SRB, originating from PG-polluted coastal marine ecosystems, to carry out such reductive process have not yet been studied.

The aim of this study was to determine the potential of SRB populations originating from marine sediments, to biotransform PG. To this end, we first evaluated the initial microbial community diversity of PG-polluted marine sediments, close to a coastal phosphate fertilizer plant, as well as that of the Tunisian PG, characterized by a low pH (~3). Marine SRB were then enriched and cultivated from PG-polluted sediments, but not directly from PG (as inoculum). Finally, we analyzed the SRB diversity of marine sediments associated with changes in H_2_S production from PG or sodium sulfate as sulfate sources using various electron donors (e.g., acetate, formate, or lactate).

## Materials and methods

### Sample collection and characteristics

PG and sediment samples were collected in March 2016 in the phosphate fertilizer complex of Sfax City, southern Tunisia. The Sfax plant (SIAPE factory) stockpiles ~30 million tons of PG 1 km inland from the coast. The PG stockpile covers more than 48 ha and measures more than 50 m high. The site area has been heavily impacted by both organic and metallic pollutants for more than 60 years (Zairi and Rouis, 1999).

Sediment samples were collected at low tide near the acid effluent discharge of the Sfax plant into the seawater (34°67.989′N, 10°74.603′E). The top 20 cm of sediment layer was sampled with a spatula and distributed in sterile plastic bags (~1 kg), and PG samples were collected from a small pile next to the largest long-term stockpile.

Both PG and marine sediment samples were stored at 4°C and aliquots were also freeze-dried and homogenized (fraction < 63 μm) before chemical analyses. Aliquots were also stored in sterile Falcon tubes at −20°C prior to DNA extraction, or stored at 4°C in a hermetically-sealed serum bottle with a nitrogen gas headspace before preparing sulfate-reducing culture enrichments.

The pH and dissolved oxygen (DO) were measured from porewater of sediment samples after transferring them into Falcon tubes and centrifugation (30 min, 10,000 × g). The pH of PG was measured after mixing it with distilled water in a ratio of 1:5 (w:v) for about 30 min. The pH and DO were determined using a pH-meter (NeoMet pH-200L) and a DO meter (Multi 3410, WTW). Aliquots of recovered waters were analyzed for sulfate content using Spectroquant tests (Merck) as per the manufacturer's instructions. For whole PG and sediment samples, water content was determined following drying for 24 h at 105°C. Total carbon, hydrogen, nitrogen, and sulfur (C/H/N/S) contents were determined in aliquots of dried PG and sediments, and total organic carbon was determined in dried and acidified PG and sediments, using a SC-144 LECO Elemental Analyzer, as described by Zaghden et al. (2017).

For the determination of total trace metals content in the sediment, digestion procedure was based on the addition of inorganic acids in a perfluoroalkoxy (PFA) closed vessel heated in a hot plate at 90°C. All acids used were concentrated and trace metalgrade (Optima, FisherChemical). A mixture of HNO_3_/HCl/HF was used to extract metals from sediments and extracts were diluted with 2% (v/v) HNO_3_ before analysis. Concentrations in trace metals (Al, As, Cd, Co, Cr, Cu, Fe, Mn, Mo, Ni, Pb, Ti, U, V, Zn) were measured by High Resolution Inductively Coupled Plasma Mass Spectrometry (HR-ICP-MS, Element XR, Thermo Scientific).

### Phosphogypsum-biotransforming and sulfate-reducing culture enrichments

Sulfate-reducing culture enrichments were performed in duplicate using 120 mL glass bottles containing the following basal medium components (g/L): NH_4_Cl (1.0), KH_2_PO_4_ (0.3), K_2_HPO_4_(0.3), NaCl (30), KCl (0.1), CaCl_2_ (0.1), MgCl_2_·6H_2_O (0.5), yeast extract (0.5), cysteine hydrochloride (0.5), and 1 mL trace mineral element solution (Widdel and Pfennig, 1982), and 1 mL 0.1% (w/v) resazurin solution. The basal culture medium was supplemented with 1.58 g/L Na_2_SO_4_ or 2 g/L PG, corresponding to the approximate solubility of PG in water (for Na_2_SO_4_ or PG cultures, respectively) acting as equivalent amount of sulfate sources, and as potential terminal electron acceptors to be used by sulfate-reducing microorganisms in culture enrichments. The initial pH was adjusted to 6.8, in order to have pH condition similar to that obtained when mixing PG and sediments with seawater. The culture basal medium was boiled and cooled down to room temperature under a continuous O_2_-free N_2_-flush. Fifty milliliters of this medium was then dispensed into glass bottles under N_2_ atmosphere and autoclaved (20 min, 120°C). Prior to inoculation, the following sterile solutions were injected in each bottle: 0.5 mL of 2.5% Na_2_S·9H_2_O (reducing agent) and 1 mL of 5% NaHCO_3_ (to adjust and buffer the pH). Different final concentrations of acetate (20 mM), lactate (40 mM), or formate (80 mM) were used as electron donors, based on the reaction stoichiometry with sulfate (acetate:sulfate = 1:1; lactate:sulfate = 2:1; formate:sulfate = 4:1). These energy sources were added separately to each bottle. Controls were also prepared without added energy sources.

The bottles were inoculated with either 5 mL of marine sediment or non-autoclaved PG (acting as inoculum source), and then incubated at 37°C for 2 weeks (with controls being prepared without inocula). Samples (100 μL) of the cultures were collected during the experiments in order to analyze H_2_S production from Na_2_SO_4_ or PG according to the method described by Cord-Ruwisch (1985). Thirty milliliters of the cultures were collected at the end of the experiments and then centrifuged (10,000 g, 10 min). The pellets and supernatants were separately stored at −20°C for further DNA extractions and chemical analysis, respectively.

The concentration of soluble end-products of metabolism was determined by high-pressure liquid chromatography (HPLC) analysis and refractometric detection (Thermo Separation Products) as previously described by Mei et al. (2014). Technical duplicate measurements were conducted on each sample. Sulfate analyses were performed by ion chromatography with chemical suppression. The chromatograph (761 Compact IC, Metrohm) was equipped with a conductivity detector and a Metrosep A Supp 1 column (Metrohm). Eluant (Na_2_CO_3_, 3 mM) was used at a flow rate of 1 to 2.5 mL/min. Twenty microliters were injected.

### DNA extraction

DNA was extracted from environmental samples (PG and sediment), as well as, duplicate enrichment cultures (sediment pellets) using the UltraClean Soil DNA Isolation Kit (MoBio Laboratories, Inc., CA), as previously described by Quéméneur et al. (2016). The choice of DNA extraction method following sampling and storage may have an impact on the revealed community structure (Luna et al., 2006). The purity and amount of extracted DNA was measured by using the Thermo Scientific Nano Drop 2000 spectrophotometer.

### 16S rRNA sequencing analysis

The mixtures of 16S rRNA gene amplicons were generated using a 341F/815R primer set, as previously described by Dowd et al. (2008), and were sequenced by the MiSeq Illumina (paired-end 2 × 300 bp) platform of the Molecular Research Laboratory (Texas, USA). Raw data were analyzed using QIIME 1.9.1 as described by Caporaso et al. (2010). Briefly, the raw reads were checked for adapter, chimera and low quality sequences. The trimmed reads were clustered into operational taxonomic units (OTUs) using a 97% sequence identity threshold with UCLUST (Edgar, 2010). The taxonomic assignment was performed by UCLUST taxonomy. Similarity search by BLAST algorithm (Altschul et al., 1990) against non-redundant nr nucleotide database was performed for OTU representative sequences. The alpha diversity Shannon and Simpson indices were also calculated. The Good's coverage was calculated according to the equation: C = 1–(n/N) where n is the number of OTU and N is the total number of sequences (Good, 1953). Sequences from selected dominant OTUs (>1% of total sequences) were aligned using Muscle (Edgar, 2004) with related sequences retrieved from NCBI databases, and a phylogenetic tree was built with MEGA7 (Kumar et al., 2016) using the Maximum Likelihood method (Tamura and Nei, 1993). Tree topology confidence was determined by bootstrap analysis on 1,000 replicates (Felsenstein, 1985). The archaeal and bacterial 16S rRNA gene reference sequences of OTUs have been deposited in the Genbank database under the accession numbers KY773181-KY773204 and KY771104-KY771161, respectively.

### Statistical analyses

Treatment effects (i.e., sulfate source, type of substrate) on parameters to be analyzed (i.e., H_2_S production, microbial diversity) were evaluated using a one-way analysis of variance (ANOVA) with a Bonferroni *post-hoc* test using SPSS version 17.0 (SPSS Statistics, Inc., Chicago, IL, USA). *P* < 0.05 were considered as statistically significant. Spearman rank correlations were used to investigate the relations among parameters (i.e., performances of H_2_S production and the proportion of individual taxon or the indices of community diversity). The data obtained at end point time of experiment (day 14) were analyzed by Principal Component Analysis (PCA) using XLSTAT (Addinsoft, XLSTAT Version 2014.5.03).

## Results and discussion

### Characteristics of marine sediments and phosphogypsum samples

The porewater of the sampled marine sediment (hereafter defined MS) had a pH-value of 6.3 and low dissolved oxygen concentration (0.27 mg/L; Table S1). MS exhibited a polymetallic contamination (Table S1). The Cd concentration (61.9 mg/kg) was 10.4-fold higher than the average concentration measured in previous coastal sediments of Sfax (7.2 ± 0.9 mg/kg), while Cr and Ni and Zn were 2.2, 2.3, and 5.3 times higher (Ghannem et al., 2010; Gargouri et al., 2011; Houda et al., 2011; Serbaji et al., 2012), indicating a metal enrichment in the MS.

The PG was characterized by a high sulfate content (55% of the PG dry weight), a very low organic carbon content (0.53 ± 0.15%), and a low pH-value (3.1), as previously reported for Tunisian PG (Azabou et al., 2005), which was lower than pH-values of European PG (Martins et al., 2016; Table S2). This PG presents various trace metals, such as Cd (21.7 ± 7.2 mg.kg^−1^) and Sr (534.6 ± 53.3 mg.kg^−1^), as previously reported in Tunisian PG (Azabou et al., 2005).

### Microbial diversity in marine sediment and phosphogypsum samples

The initial microbial diversity was evaluated in both MS and PG samples (Table 1). With regard to the alpha diversity, the MS showed higher bacterial species richness (observed OTU) and species diversity (Shannon, Simpson indices) than the PG (Table 1).

**Table 1:** Richness and diversity of microbial communities in marine sediment (MS) and phosphogypsum (PG) samples of Sfax (Tunisia), and in the sulfate-reducing enrichment cultures from MS (as inoculum) using sodium sulfate or phosphogypsum (PG) as sulfate source.

**Samples**	**Sequences**	**OTUs**	**OTUs > 1%**	**Chao1**	**Simpson**	**Shannon**
MS	88799	1686	8	1827.3	0.99	8.98
PG	35018	224	20	275.8	0.88	4.51
SF	99257.5 ± 29925.5	428 ± 18	16.5 ± 1.5	472.0 ± 6.5	0.91 ± 0.01	4.96 ± 0.04
SL	124618.5 ± 47462.5	488.5 ± 11.5	18.5 ± 2.5	519.4 ± 7.9	0.92 ± 0.01	5.57 ± 0.22
SA	114070 ± 13716	461.0 ± 5.0	12.5 ± 1.5	506.3 ± 3.1	0.87 ± 0.01	4.62 ± 0.10
SPF	152322 ± 7878	499.5 ± 18.5	8.0 ± 3.0	521.9 ± 4.3	0.59 ± 0.09	3.06 ± 0.42
SPL	138723 ± 21477	473.5 ± 44.5	12.0 ± 1.0	533.3 ± 17.9	0.79 ± 0.08	3.93 ± 0.50
SPA	111330.5 ± 4144.5	491.5 ± 9.5	14.5 ± 0.5	513.3 ± 9.8	0.92 ± 0.02	5.02 ± 0.23

*The names of enrichment cultures have been abbreviated as follows: SF, SL, and SA, for Na_2_SO_4_ enrichment cultures with formate, lactate, and acetate as electron donors, respectively; SPF, SPL, and SPA, for PG enrichment cultures with formate, lactate, and acetate as electron donors, respectively. Values are means of two biological replicates ± confidence intervals (error bars)*.

**Figure 1:**
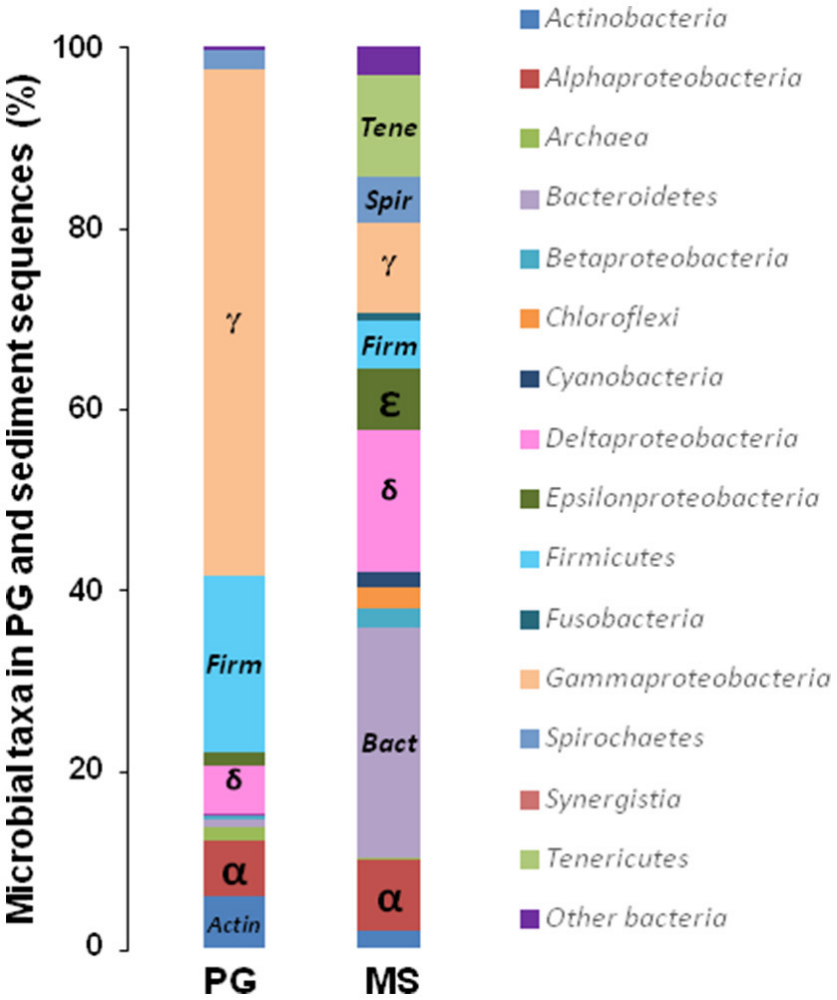
Compositions of microbial communities in the coastal marine sediment and phosphogypsum samples of Sfax (Tunisia). Relative phylogenetic abundance was based on frequencies of 16S rRNA gene sequences affiliated with *Archaea* and major bacterial phyla or proteobacterial classes in the microbial communities of marine sediment (MS) and phosphogypsum (PG).

In MS, the OTUs were mainly assigned to 9 bacterial phyla: *Proteobacteria* (42.2% of the sequences), *Bacteroidetes* (25.5%), *Tenericutes* (11.2%), *Spirochaetes* (5.0%), *Chloroflexi* (2.5%), *Actinobacteria* (2.0%), *Cyanobacteria* (1.6%), *Fusobacteria* (0.8%; Figure 1). Interestingly, MS was dominated by only 8 OTUs (>1% of all sequences), related to *Proteobacteria, Bacteroidetes, Tenericutes* phyla (Table S3). Among the *Proteobacteria, Deltaproteobacteria* sequences accounted for 15.6% of the total sequences. Two abundant deltaproteobacterial OTUs were closely related to *Desulfobacter latus* (3.2% of the sequences) and *Desulfocella halophila* (2.7%). These SRB species have been isolated from saline and marine environments (Widdel, 1987; Brandt et al., 1999). *Archaea* accounted for only 0.12% of the microbial communities and were only represented by five potentially hydrogenotrophic methanogenic OTUs (Table S4).

The PG microbial composition was almost exclusively dominated by *Proteobacteria* (>70%) and *Firmicutes* (~20%; Figure 1). Among the sixteen abundant OTUs (>1%), two *Deltaproteobacteria* OTUs were affiliated to the genera *Desulfovibrio* (1.55%), and *Desulfobacterium* (1.74%; Table S5). These results demonstrated a shift in SRB community structure presumably driven by the extreme PG characteristics (i.e., acidic pH ~3, high contents of metals, low total organic carbon). The sulfur-oxidizing *Sulfurovum lithotrophicum* (Inagaki et al., 2004), also detected as dominant species in MS, represented 1.5% of the PG community, suggesting that it was inherent to PG and that oxidation-reduction of sulfurous compounds may occur in the extreme conditions prevailing in PG. *Archaea* in PG accounted for 1.53% of the microbial community and were mainly affiliated with the *Euryarchaeota* phylum (Table S6). The most abundant archaeal OTUs from PG were distantly related to *Methanomassiliicoccus luminyensis*, reported as a hydrogenotrophic methanogen using methanol as terminal electron acceptor (Dridi et al., 2012). *M. luminyensis* were also detected in low-pH environments impacted by acid mine drainage (Mendez-Garcia et al., 2014).

### Microbial H_2_S production using sodium sulfate or phosphogypsum as sulfate source

**Figure 2:**
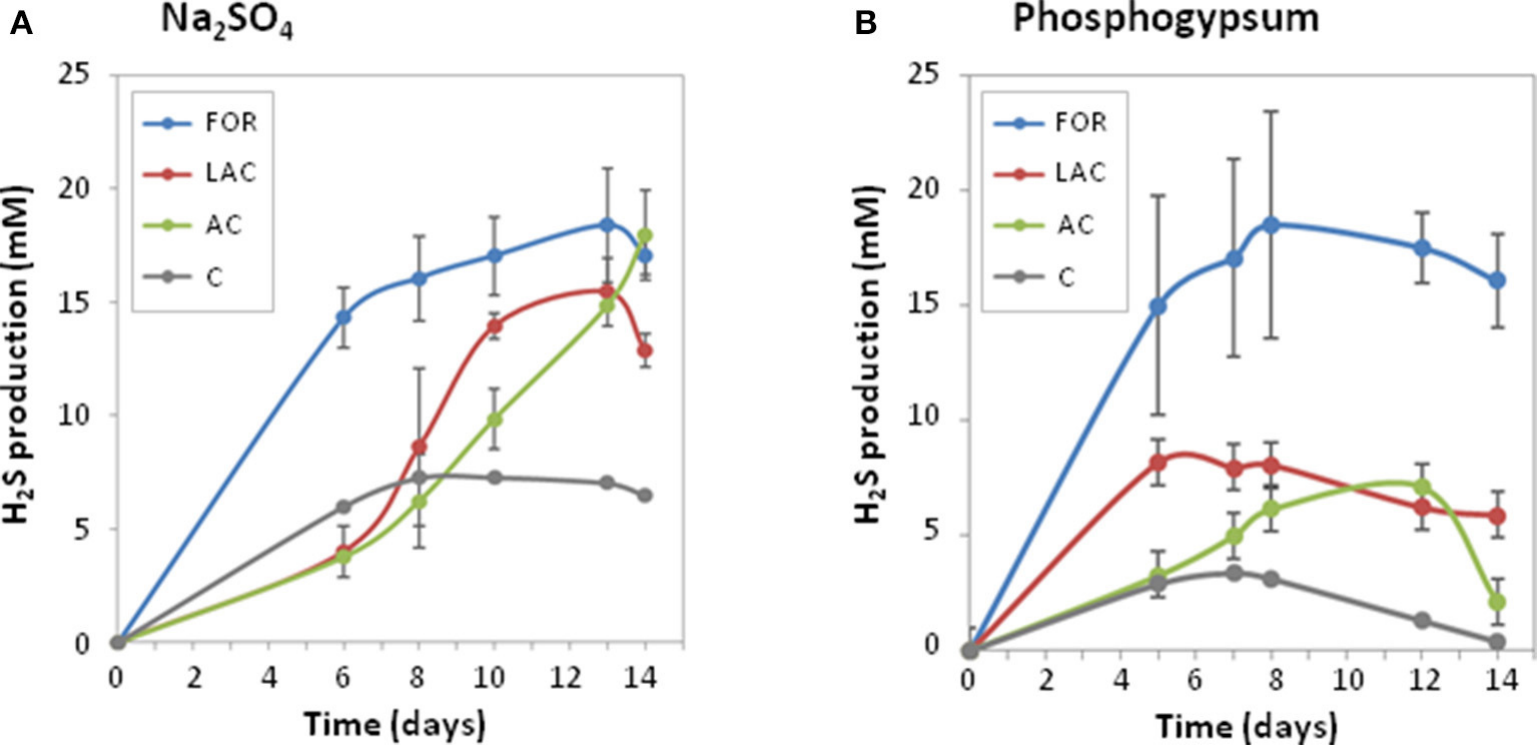
Profiles of hydrogen sulfide production during 14 days of enrichment cultures from coastal marine sediment (as inoculum) using different electron donors (AC, acetate; FOR, formate; LAC, lactate; C, control without electron donor) and sulfate sources: sodium sulfate **(A)** or phosphogypsum **(B)**. Values are means of two biological replicates ± confidence intervals (error bars).

During the 14-day experiment, H_2_S was produced by the MS microbial community using Na_2_SO_4_ or PG as sulfate sources (Figure 2), indicating that SRB originating from MS can efficiently use PG. As expected, low H_2_S production was obtained in MS controls without addition of electron donors. No H_2_S production was observed in controls without MS inoculum or with PG only, acting as inoculum and sulfate sources, indicating that SRB present in PG were not enriched in these conditions and most probably preferred acidic conditions for growth. The microbial cultures enriched from MS were then further analyzed for H_2_S production performances and microbial community compositions.

The type of added substrate (AC, acetate; FOR, formate; LAC, lactate) in the culture medium had a significant effect on H_2_S production rates (*p* < 0.005; Figure 2). Among the three different kinds of electron donors tested, FOR gave the highest H_2_S production rates whatever the sulfate source (2.4 ± 0.2 mM/day with Na_2_SO_4_ or 3.0 ± 0.2 mM/day with PG), while low H_2_S production rates were observed with AC (<1 mM/day; Table 2). In Na_2_SO_4_ cultures, no significant difference in maximum H_2_S production was observed using different electron donors (Table 2). In PG cultures, the highest maximum H_2_S production was observed using FOR (19.7 ± 3.7 mM), while oxidation of AC and LAC led to a 2 times lower H_2_S production than FOR. This result suggests that PG inhibited growth of some AC- and LAC-utilizing SRB, and/or favored incomplete SRB.

**Table 2:** Production of sulfide and consumption of electron donors and sulfate in the sulfate-reducing enrichment cultures from marine sediment (as inoculum) using sodium sulfate or phosphogypsum as sulfate source.

**Enrichment cultures**	**P_max_ sulfide (mM)**	**V_max_ sulfide (mM/day)**	**P_max_ time sulfide (day)**	**Final pH**	**Electron donor consumed (%)**	**Sulfate consumed (%)**
SF	18.6 ± 2.3	2.4 ± 0.2	10.0 ± 2.0	8.5 ± 0.1	100.0 ± 0.0	70.3 ± 21.2
SL	15.5 ± 1.5	0.7 ± 0.2	9.5 ± 2.5	7.4 ± 0.1	100.0 ± 0.0	91.3 ± 8.5
SA	17.9 ± 2.0	0.6 ± 0.0	12.0 ± 0.0	7.5 ± 0.1	81.6 ± 18.4	97.9 ± 1.5
SPF	19.7 ± 3.7	3.0 ± 1.0	10.0 ± 2.0	7.6 ± 0.0	100.0 ± 0.1	87.5 ± 6.5
SPL	8.7 ± 1.7	1.6 ± 0.3	7.5 ± 0.5	6.2 ± 0.0	67.5 ± 17.5	89.5 ± 6.5
SPA	7.5 ± 1.7	0.7 ± 0.2	10.0 ± 2.0	5.5 ± 0.0	95.0 ± 5.0	32.0 ± 4.0

*The names of enrichment cultures have been abbreviated as follows: SF, SL, and SA, for Na_2_SO_4_ enrichment cultures with formate, lactate, and acetate as electron donors, respectively; SPF, SPL, and SPA, for PG enrichment cultures with formate, lactate, and acetate as electron donors, respectively. Values are means of two biological replicates ± confidence intervals (error bars)*.

**Figure 3:**
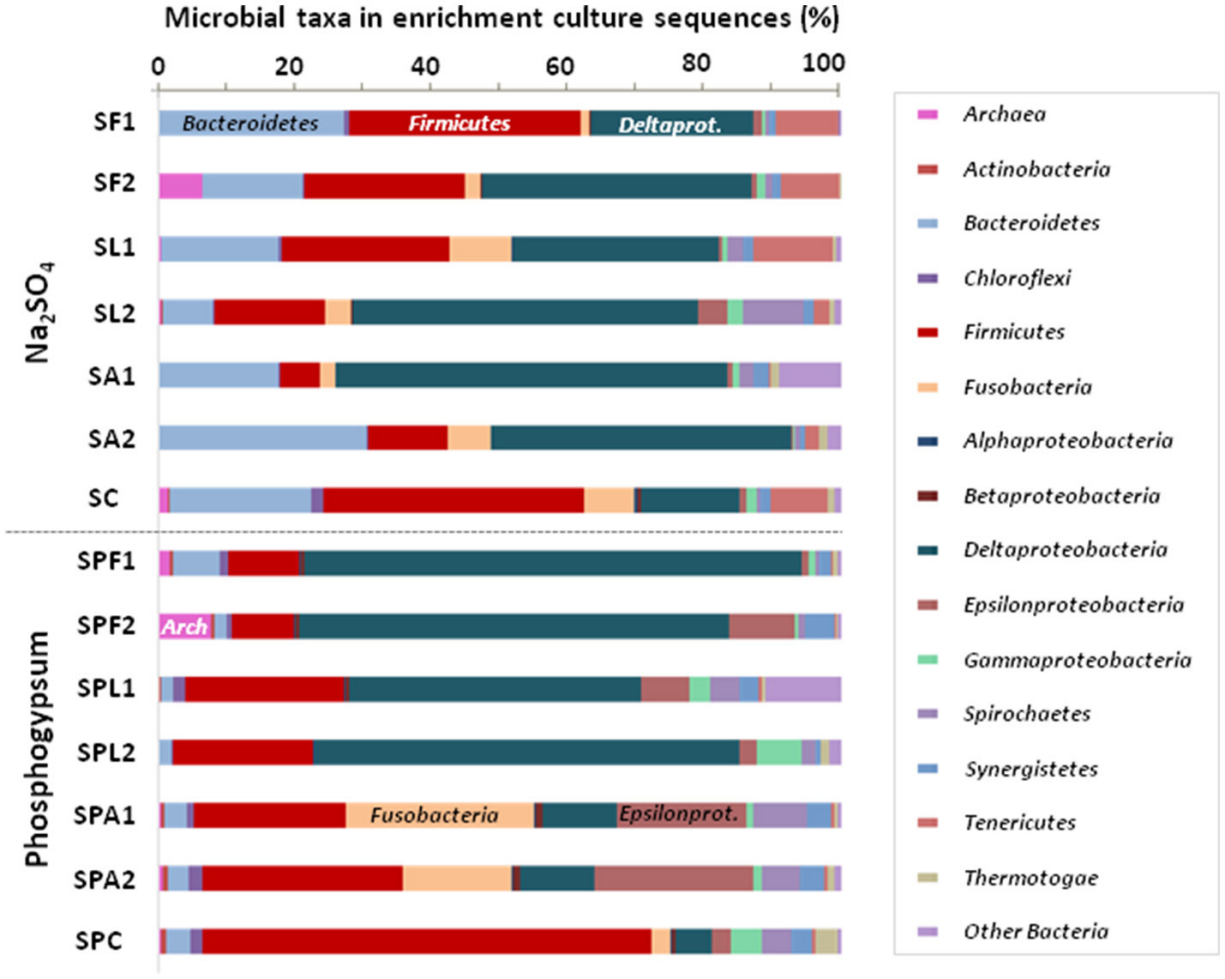
Compositions of microbial communities in sulfate-reducing enrichment cultures from marine sediment using different electron donors and sulfate sources after 14 days. Relative phylogenetic abundance was based on frequencies of 16S rRNA gene sequences affiliated with *Archaea* and major bacterial phyla or proteobacterial classes in the microbial communities. The names of enrichment cultures (duplicates 1 and 2) have been abbreviated as follows: SF, SL, SA, and SC for Na_2_SO_4_ enrichment cultures with formate, lactate, acetate and without electron donor, respectively; SPF, SPL, SPA, and SPC for PG enrichment cultures with formate, lactate, acetate, and without electron donor, respectively.

During sulfate reduction, pH increased with increasing H_2_S production (Table 2). A significant and positive correlation was observed between final pH and maximum H_2_S production (*r* = 0.91, *p* < 0.05). FOR was completely consumed in both Na_2_SO_4_ and PG cultures. In Na_2_SO_4_ cultures, LAC was incompletely oxidized into AC and propionate, indicating that SRB and other metabolic groups were involved. On the contrary, LAC was mainly converted into AC in PG cultures, indicating that incompletely oxidizing SRB were most likely involved. The majority of AC was oxidized either in the presence of Na_2_SO_4_ or that of PG (81.6 ± 18.4% and 95.0 ± 5.0%, respectively), despite the difference in sulfate reduction (97.9 ± 1.5% and 32.0 ± 4.0% of sulfate consumed, respectively; Table 2). This result suggests that PG may inhibit the growth of some AC-oxidizing SRB, such as *Desulfobacter* spp. In contrast, PG may promote growth of other acetotrophs pertaining to the *Epsilonproteobacteria* (Figure 3), such as *Arcobacter* spp. (Hubert et al., 2012; Roalkvam et al., 2015).

### Microbial diversity in the enrichment cultures containing phosphogypsum or sodium sulfate

The microbial diversity of enrichment cultures was evaluated at the end of the experiment (day 14). After filtering, the reads were normalized to 69,332 for the different cultures. The number of OTUs in each culture varied from 410 to 518 (Table 1). The sample coverage were similar for all cultures (>99%), indicating a good description of the total microbial communities. The type of sulfate source (PG or Na_2_SO_4_) had a significant effect on Shannon diversity index (*p* < 0.05; Table 1).

Both Na_2_SO_4_ and PG cultures were dominated by *Deltaproteobacteria* (42.3 ± 16.1% of the microbial communities) and *Firmicutes* (19.2 ± 7.1%; Figure 3). *Deltaproteobacteria* were abundant in the majority of cultures, except in AC + PG cultures, which displayed the lowest H_2_S production, and where they represented only 10.9% of the total communities. These AC + PG cultures mainly consisted of *Firmicutes* (25.8 ± 3.6%), followed by *Fusobacteria* (21.6 ± 5.8%), *Epsilonproteobacteria* (21.1 ± 2.2%), and *Spirochaetes* (6.6 ± 1.2%). *Firmicutes* were also abundant in LAC + PG cultures (21.8 ± 1.3%), but they were found in small proportion in efficient FOR + PG cultures. *Bacteroidetes* were also retrieved in low proportion in PG cultures (3.1 ± 1.9%), while they represented 19.0 ± 8.3% in Na_2_SO_4_ cultures, indicating that *Bacteroidetes* species can be inhibited by PG.

Despite sulfide was produced, methanogenic *Archaea* were also detected in FOR cultures (Figure 3). In Na_2_SO_4_ cultures, they comprised marine or halotolerant hydrogenotrophic belonging to the genera *Methanococcus* and *Methanocalculus* (99% identity; Ollivier et al., 1998; Table S7), while they were closely related to the marine and hydrogenotrophic *Methanogenium marinum* (99% identity; Chong et al., 2002) in PG cultures (Table S7).

**Figure 4:**
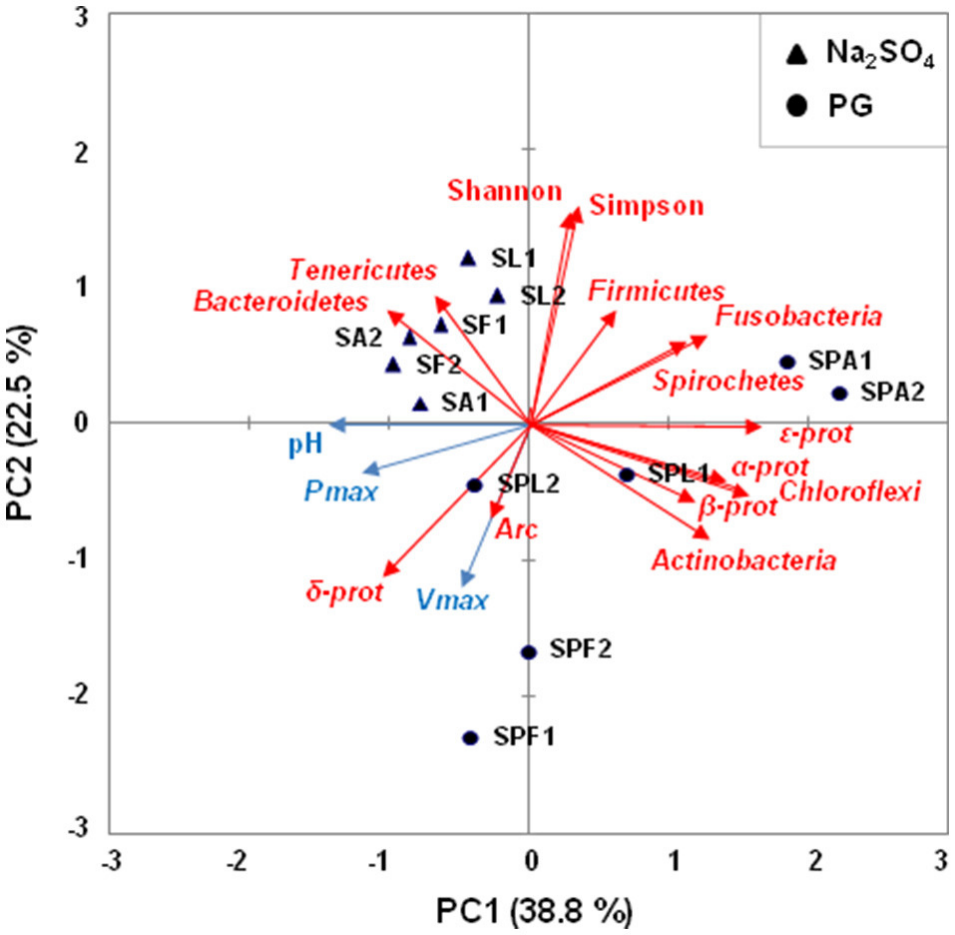
Principal Component Analysis (PCA) biplot showing the variation among the enrichment cultures based on hydrogen sulfide production performances and the relative abundance of microbial taxa. Black circles represent PG enrichment cultures and black triangles represent Na_2_SO_4_ enrichment cultures. The names of enrichment cultures (duplicates 1 and 2) have been abbreviated as follows: SF, SL, and SA, for Na_2_SO_4_ enrichment cultures with formate, lactate, and acetate as electron donors, respectively; SPF, SPL, and SPA, for PG enrichment cultures with formate, lactate, and acetate as electron donors, respectively. Arrows indicate the direction of maximum increase and strength (through the length) of each variable to the overall distribution. The blue arrows are indicators of hydrogen sulfide production (pH, P_max_, V_max_) and the red arrows represent the microbial taxa. Among these latter, α-Prot, β-Prot, δ-Prot, ε-Prot stand for *Alphaproteobacteria, Betaproteobacteria, Deltaproteobacteria*, and *Epsilonproteobacteria*. The first two principal axes explained 61.3% of the variance.

A PCA was performed to identify the factors that affect the microbial community at the end of the experiment (Figure 4). The first two principal components explained 61.3% of the variability in the data. A Spearman's rank correlation analysis was also used to examine the relationships between the microbial diversity and the PG biotransformation through three factors of H_2_S production performances (i.e., final pH, P_max_, and V_max_; Table S8). As expected, significant and positive correlations were observed between the *Deltaproteobacteria* proportions and the final pH (*r* = 0.94, *p* < 0.05), P_max_ (*r* = 0.83, *p* < 0.05), and V_max_ (*r* = 0.89, *p* < 0.05). In contrast, the proportions of *Firmicutes, Fusobacteria*, as well as those of *Spirochaetes*, were negatively correlated with the H_2_S production performances. The values of Simpson index were also significantly and negatively correlated with the final pH (*r* = −0.94, *p* < 0.05), P_max_ (*r* = −0.83, *p* < 0.05), and V_max_ (*r* = −0.89, *p* < 0.05), indicating that PG transformation was associated with low microbial diversity.

### Dominant bacterial species in the enrichment cultures containing phosphogypsum or sodium sulfate

The number of abundant OTUs (>1%) was very low in SRB enrichment cultures (Table 1), but they accounted for 60.7–86.1% of all sequences.

**Figure 5:**
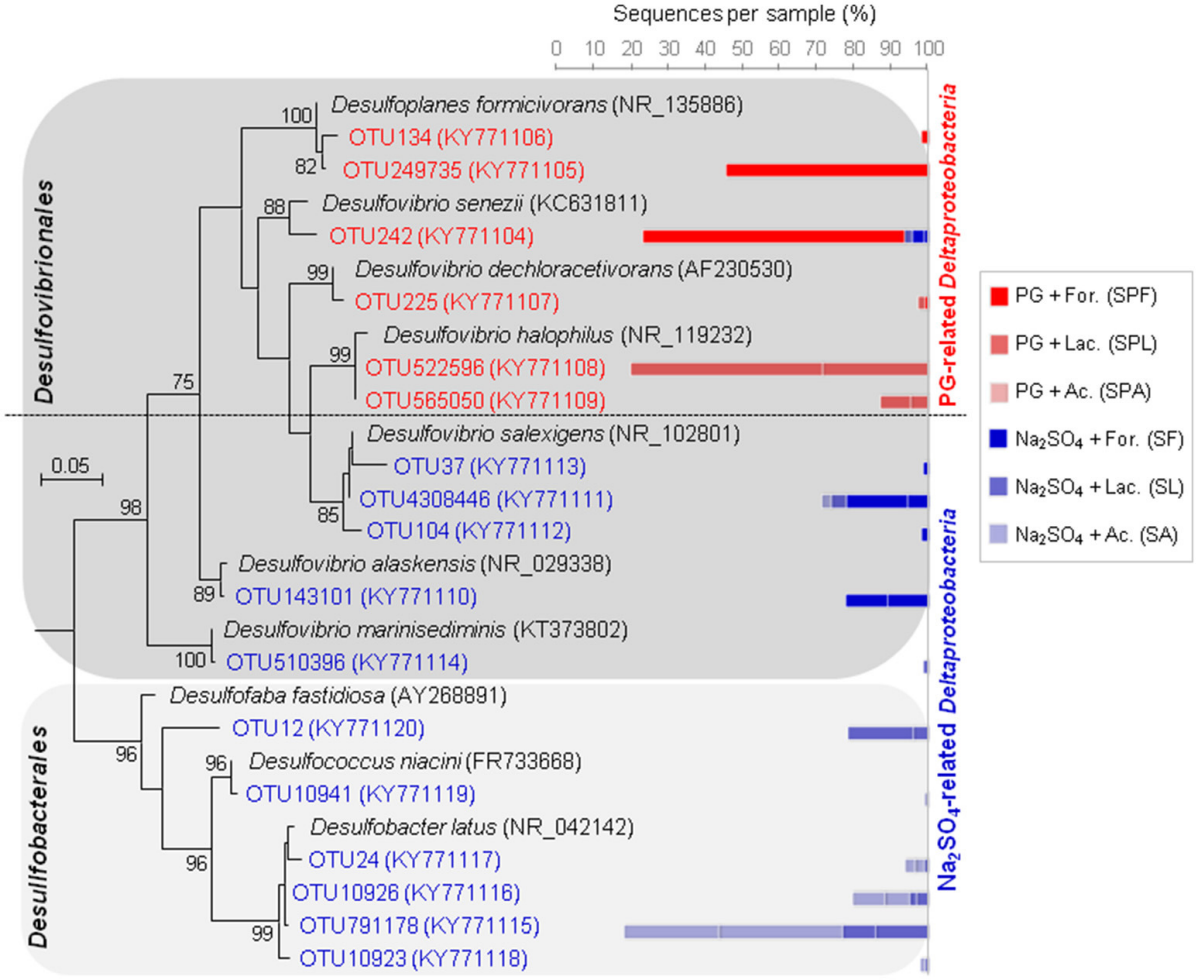
Maximum-likehood (ML) tree based on 16S rRNA gene sequences showing the phylogenetic position of *Deltaproteobacteria* enriched from marine sediment (as inoculum) using different electron donors and sulfate sources (sodium sulfate or phosphogypsum). Representative sequences in the tree were obtained from GenBank (accession number in the brackets). Bootstrap values >75% are indicated at nodes. The bars represent the relative abundance of each OTU affiliated with *Deltaproteobacteria* in the enrichment cultures. The blue bars indicate the relative abundance of OTUs in sodium sulfate cultures, whereas the red bars represent the relative abundance of OTUs in PG cultures.

These abundant OTUs were mainly assigned to two *Deltaproteobacteria* groups (Figure 5). The first deltaproteobacterial group present in PG cultures comprised *Desulfovibrio* spp., which are known as powerful hydrogenotrophic SRB. It was found in high proportions in both FOR and LAC + PG cultures. The highest H_2_S-producing FOR + PG cultures were dominated by two OTUs phylogenetically related to halotolerant sulfate-, thiosulfate- and sulfite-reducing species, *Desulfovibrio senezii* (94% identity) and *Desulfoplanes formicivorans* (98% identity), which use FOR and LAC as electron donors (Tsu et al., 1998; Watanabe et al., 2015). In contrast, LAC + PG cultures were dominated by SRB having 98% identity with *Desulfovibrio halophilus*, a halophilic SRB oxidizing LAC to AC (Caumette et al., 1991). In Na_2_SO_4_ cultures, FOR cultures were also dominated by halophilic *Desulfovibrio* spp. The second deltaproteobacterial group containing *Desulfobacter* spp. (Figure 5), which are active AC-oxidizing SRB (contrary to *Desulfovibrio* spp.), was only retrieved in Na_2_SO_4_ cultures, suggesting that such members were inhibited by PG. The absence of *Desulfobacter* spp. in PG cultures could be explained by the presence of trace metals in PG. Depending on SRB species, the toxic trace metal concentrations reported for SRB generally ranged from a few mg/L to as much as 100 mg/L (Utgikar et al., 2002; Azabou et al., 2007b). For instance, Zn can be toxic at concentrations varying between 25 and 150 mg/L, while toxic Cd concentration can range between 4 and 40 mg/L (Hao et al., 1994; Azabou et al., 2007a; Martins et al., 2009).

**Figure 6:**
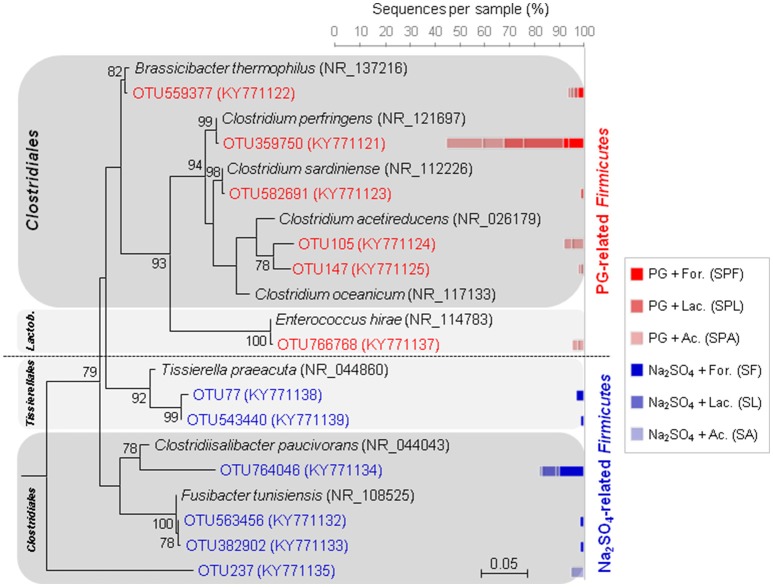
Maximum-likehood (ML) tree based on 16S rRNA gene sequences showing the phylogenetic position of *Firmicutes* from marine sediment (as inoculum) using different electron donors and sulfate sources (sodium sulfate or phosphogypsum). Representative sequences in the tree were obtained from GenBank (accession number in the brackets). Bootstrap values >75% are indicated at nodes. The bars represent the relative abundance (in %) of each OTU affiliated with *Firmicutes* in the enrichment cultures. The blue bars indicate the relative abundance of OTUs in sodium sulfate cultures, whereas the red bars represent the relative abundance of OTUs in PG cultures.

The abundant populations of *Firmicutes* also changed depending on sulfate sources. In PG cultures, the majority of abundant *Firmicutes* were related to clostridial species (Figure 6), such as the non-sulfate-reducing bacterium, *Clostridium perfringens*, which may use thiosulfate and sulfite as electron acceptors (Fuchs and Bonde, 1957; André et al., 2010), or the potential sulfate, thiosulfate- or sulfite-reducer, *Brassicibacter thermophilus* (Wang et al., 2015). Contrary to PG cultures, the Na_2_SO_4_ cultures were represented by a higher number of haloterant or halophilic genera also affiliated to the *Clostridiales* order (Figure 6), such as the thiosulfate- and/or sulfur-reducing *Fusibacter* spp. (Ben Hania et al., 2012) and the non-sugar fermenting *Clostridiisalibacter* spp. (Liebgott et al., 2008), isolated from olive mill wastewater in Tunisia. The absence of these clostridial species in PG cultures may result from toxicity of PG or may due to competition with other bacterial species in its presence.

## Conclusions

This study demonstrates that MS microbial communities, cultivated in microcosms under laboratory conditions and fed with different electron donors, can efficiently convert sulfate contained in PG into H_2_S. The type of electron donor to be used in such process was found as key criterion influencing H_2_S production from enriched microbial communities. Whatever the sulfate source (PG or Na_2_SO_4_), formate led to more efficient H_2_S production performances when compared to acetate and lactate. The differences observed in H_2_S production performances using diverse electron donors was mainly related to the enrichment of specific deltaproteobacterial species. Among *Deltaproteobacteria, Desulfovibrio* species were found to be the most efficient SRB to bioremediate PG by producing H_2_S, while *Desulfobacter* species, which were found only abundant in Na_2_SO_4_ cultures, seemed to be inhibited by PG. Finally, we showed that the emergence of some clostridial species, as well as *Spirochaetes* members, in sulfate enrichment cultures could have a negative consequence on H_2_S production from PG. Further work to isolate SRB from marine sediments, especially *Desulfovibrionales* species is needed, to test their metal resistance and their performance in PG biotransformation.

## Author contributions

HZ, FK, and MQ designed experiments. HZ performed experiments and analyzed data. FA, SC, AH, WB, and MQ helped for data acquisition. MQ wrote the manuscript in collaboration with HZ. All authors read and commented on the draft manuscript. All authors agreed to the final version.

### Conflict of interest statement

The authors declare that the research was conducted in the absence of any commercial or financial relationships that could be construed as a potential conflict of interest.
