# Supplementary material for: Microbial Diversity in Sulfate-Reducing Marine Sediment Enrichment Cultures Associated with Anaerobic Biotransformation of Coastal Stockpiled Phosphogypsum (Sfax, Tunisia)
Source: Front Microbiol. 2017 Aug 21;8:1583. doi: 10.3389/fmicb.2017.01583 (PMC5566975; doi:10.3389/fmicb.2017.01583)
Supplement: Supplementary file 1 [file Table1.DOCX]

**Table S1. Chemical properties of the marine sediment (MS) sample collected on the south coast of Sfax (Tunisia).**

|  | MS |
| --- | --- |
| pH^1^ | 6.25 |
| Dissolved oxygen (mg/L)^1^ | 0.27 |
| Carbon (%) | 5.25 |
| Organic carbon (%) | 4.65 |
| Hydrogen (%) | 0.31 |
| Nitrogen (%) | 1.12 |
| Sulfur (%) | 1.10 |
| Water content (%) | 11.78 |
| Trace metals (mg/kg) | |
| As | 4.8 |
| Cd | 61.9 |
| Co | 1.3 |
| Cr | 106.2 |
| Cu | 29.9 |
| Fe | 4715.5 |
| Mn | 74.1 |
| Mo | 5.0 |
| Ni | 44.4 |
| Pb | 39.7 |
| Sb | 0.7 |
| Ti | 1173.0 |
| U | 20.4 |
| V | 23.6 |
| Zn | 448.8 |

^1^ Values obtained from porewater extracted from MS

^2^ Percentage value for 1 g sediment dry weight
