# Supplementary material for: Microbial Diversity in Sulfate-Reducing Marine Sediment Enrichment Cultures Associated with Anaerobic Biotransformation of Coastal Stockpiled Phosphogypsum (Sfax, Tunisia)
Source: Front Microbiol. 2017 Aug 21;8:1583. doi: 10.3389/fmicb.2017.01583 (PMC5566975; doi:10.3389/fmicb.2017.01583)
Supplement: Supplementary file 2 [file Table2.DOCX]

**Table S2. Composition of phosphogypsum from Sfax (Tunisia)**

|  | PG |
| --- | --- |
| pH | 3.05 |
| Water content (%) | 14 |
| SO_4_ (%)^1^ | 55 |
| Carbon (%)^1, 2^ | 0.53 ± 0.15 |
| Organic carbon (%)^1, 2^ | 0.53 ± 0.15 |
| Hydrogen (%)^1, 2^ | < 0.05 |
| Nitrogen (%)^1, 2^ | 2.20 ± 0.02 |
| Sulfur (%)^1, 2^ | 7.23 ± 2.40 |
| Trace metals (mg/kg)^2^ | |
| As | 1.8 ± 0.5 |
| Cd | 21.7 ± 7.2 |
| Co | 0.4 ± 0.2 |
| Cu | 5.4 ± 1.9 |
| Mo | 2.4 ± 1.4 |
| Ni | 6.8 ± 3.4 |
| Pb | 1.0 ± 0.5 |
| Sb | 0.2 ± 0.1 |
| Sr | 534.6 ± 53.3 |
| U | 12.2 ± 10.7 |
| V | 19.0 ±16.2 |
| Zn | 166.6 ± 53.8 |

^1^ Percentage values for 1 g PG dry weight.

^2^ Values are means of three PG subsamples of Tunisian Chemical Group ± confidence intervals (error bars).
