# Supplementary material for: Microbial Diversity in Sulfate-Reducing Marine Sediment Enrichment Cultures Associated with Anaerobic Biotransformation of Coastal Stockpiled Phosphogypsum (Sfax, Tunisia)
Source: Front Microbiol. 2017 Aug 21;8:1583. doi: 10.3389/fmicb.2017.01583 (PMC5566975; doi:10.3389/fmicb.2017.01583)
Supplement: Supplementary file 3 [file Table3.DOCX]

**Table S3. Blast analysis on the dominant OTUs (>1% of total sequences) obtained from microbial communities in marine sediment (MS) sample of Sfax (Tunisia).**

| OTU no. [GenBank number] | Sequences per sample (%) | Closest cultivated relative retrieved from NCBI nucleotide database | | |
| --- | --- | --- | --- | --- |
|  |  | Taxonomy (Phylum/class; order) | Species [accession number] | Identity (%) |
| 4390055 [KY771159] | 1.4 | *Alphaproteobacteria; Rhodobacterales* | *Thioclava indica* [NR_136454] | 99 |
| 791178 [KY771115] | 3.2 | *Deltaproteobacteria; Desulfobacterales* | *Desulfobacter latus* [AJ441315] | 97 |
| 40 [KY771156] | 2.7 | *Deltaproteobacteria; Desulfobacterales* | *Desulfocella halophila* [NR_024858] | 95 |
| 153118[KY771147] | 1.0 | *Epsilonproteobacteria* | *Sulfurovum lithotrophicum* [CP011308] | 97 |
| 560122 [KY771160] | 1.1 | *Bacteroidetes; Bacteroidales* | *Draconibacterium orientale* [KF041475] | 99 |
| 394758 [KY771157] | 1.8 | *Bacteroidetes; Flavobacteriales* | *Winogradskyella litoriviva* [NR_137338] | 99 |
| 14 [KY771161] | 1.1 | *Tenericutes* | *Candidatus Izimaplasma* [CP009415] | 93 |
| 1157 [KY771158] | 1.4 | *Tenericutes* | *Candidatus Izimaplasma* [CP009415] | 86 |
