# Supplementary material for: Microbial Diversity in Sulfate-Reducing Marine Sediment Enrichment Cultures Associated with Anaerobic Biotransformation of Coastal Stockpiled Phosphogypsum (Sfax, Tunisia)
Source: Front Microbiol. 2017 Aug 21;8:1583. doi: 10.3389/fmicb.2017.01583 (PMC5566975; doi:10.3389/fmicb.2017.01583)
Supplement: Supplementary file 4 [file Table4.DOCX]

**Table S4. Blast analysis on the archaeal OTUs obtained from the marine sediment sample (MS) of Sfax (Tunisia).**

| OTU no.  [GenBank number] | Sequences (%) | Closest cultivated relative retrieved from NCBI nucleotide database | | |
| --- | --- | --- | --- | --- |
|  |  | Taxonomy (Phylum ; class) | Species [accession number] | Identity (%) |
| 826668 [KY773200] | 0.057 | *Eury ; Methanomicrobia* | *Methanosaeta concilii* [NR_102903] | 99 |
| 561472 [KY773203] | 0.017 | *Eury ; Methanomicrobia* | *Methanosaeta harundinacea* [NR_043203] | 98 |
| 15646 [KY773201] | 0.026 | *Eury ; Methanomicrobia* | *Methanospirillum hungatei* [NR_074177] | 98 |
| 814666 [KY773204] | 0.003 | *Eury ; Methanobacteria* | *Methanothermus fervidus* [NR_102926] | 77 |
| 3388 [KY773202] | 0.020 | *Eury ; Methanococci* | *Methanothermococcus okinawensis* [NR_102915] | 79 |
