# Supplementary material for: Microbial Diversity in Sulfate-Reducing Marine Sediment Enrichment Cultures Associated with Anaerobic Biotransformation of Coastal Stockpiled Phosphogypsum (Sfax, Tunisia)
Source: Front Microbiol. 2017 Aug 21;8:1583. doi: 10.3389/fmicb.2017.01583 (PMC5566975; doi:10.3389/fmicb.2017.01583)
Supplement: Supplementary file 5 [file Table5.DOCX]

**Table S5. Blast analysis on the dominant OTUs (>1% of total sequences) obtained from microbial communities in phosphogypsum (PG) sample of Sfax (Tunisia).**

| OTU no. [GenBank number] | Sequences per sample (%) | Closest cultivated relative retrieved from NCBI nucleotide database | | |
| --- | --- | --- | --- | --- |
|  |  | Taxonomy (phylum/classe ; order) | Species (accession number) | Identity (%) |
| 574655 [KY771142] | 2.6 | *Alphaproteobacteria; Rhizobiales* | *Methylobacterium phyllosphaerae* [CP015367] | 99 |
| 68458 [KY771148] | 1.3 | *Alphaproteobacteria; Rhizobiales* | *Methylobacterium jeotgali* [NR_043878] | 99 |
| 46 [KY771145] | 1.7 | *Alphaproteobacteria; Rhodobacterales* | *Albimonas donghaensis* [NR_043685] | 95 |
| 14988 [KY771151] | 1.7 | *Deltaproteobacteria; Desulfobacterales* | *Desulfobacterium vacuolatum* [AF418178] | 95 |
| 4261 [KY771152] | 1.6 | *Deltaproteobacteria; Desulfovibrionales* | *Desulfovibrio dechloracetivorans* [KJ576635] | 99 |
| 153118 [KY771147] | 1.5 | *Epsilonproteobacteria* | *Sulfurovum lithotrophicum* [CP011308] | 98 |
| 646549 [KY771140] | 8.4 | *Gammaproteobacteria; Pseudomonadales* | *Pseudomonas yamanorum* [LT673850] | 99 |
| 216324 [KY771141] | 4.9 | *Gammaproteobacteria; Pseudomonadales* | *Acinetobacter johnsonii* [LT160758] | 99 |
| 60 [KY771146] | 1.5 | *Gammaproteobacteria; Oceanospirillales* | *Salinicola salarius* [KP178609] | 98 |
| 1581[KY771149] | 1.3 | *Gammaproteobacteria; Cellvibrionales* | *Halioglobus pacificus* [NR_113279] | 96 |
| 6708 [KY771150] | 1.2 | *Gammaproteobacteria; Chromatiales* | *Halochromatium salexigens* [NR_036810] | 91 |
| 1002005 [KY771143] | 2.2 | *Actinobacteria; Micrococcales* | *Kocuria palustris* [KX108883] | 99 |
| 4386514 [KY771153] | 9.1 | *Firmicutes; Bacillales* | *Bacillus licheniformis* [HG800002] | 99 |
| 78 [KY771154] | 2.3 | *Firmicutes; Bacillales* | *Bacillus licheniformis* [HG800002] | 99 |
| 404204 [KY771155] | 2.3 | *Firmicutes; Bacillales* | *Bacillus licheniformis* [HG800003] | 99 |
| 13344 [KY771144] | 2.1 | *Spirochaetes; Spirochaetales* | *Spirochaeta cellobiosiphila* [NR_044505] | 87 |
