# Supplementary material for: Microbial Diversity in Sulfate-Reducing Marine Sediment Enrichment Cultures Associated with Anaerobic Biotransformation of Coastal Stockpiled Phosphogypsum (Sfax, Tunisia)
Source: Front Microbiol. 2017 Aug 21;8:1583. doi: 10.3389/fmicb.2017.01583 (PMC5566975; doi:10.3389/fmicb.2017.01583)
Supplement: Supplementary file 6 [file Table6.DOCX]

**Table S6. Blast analysis on the archaeal OTUs obtained from the phosphogypsum (PG) of Sfax (Tunisia).**

| OTU no.  [GenBank number] | Sequences (%) | Closest cultivated relative retrieved from NCBI nucleotide database | | |
| --- | --- | --- | --- | --- |
|  |  | Taxonomy (Phylum ; class) | Species [accession number] | Identity (%) |
| 11486 [KY773193] | 0.957 | *Eury ; Thermoplasmata* | *Methanomassiliicoccus luminyensis* [NR_118098] | 83 |
| 782932 [KY773198] | 0.003 | *Eury ; Thermoplasmata* | *Methanomassiliicoccus luminyensis* [NR_118098] | 83 |
| 202345 [KY773199] | 0.003 | *Eury ; Thermoplasmata* | *Methanomassiliicoccus luminyensis* [NR_118098] | 83 |
| 553907 [KY773194] | 0.348 | *Eury ; Halobacteria* | *Halorubrum coriense* [NR_113474] | 99 |
| 6448 [KY773197] | 0.006 | *Eury ; Methanomicrobia* | *Methanogenium marinum* [NR_02822] | 98 |
| 3489142 [KY773195] | 0.197 | *Eury ; Methanomicrobia* | *Methanosalsum natronophilum* [NR_137354] | 72 |
| 18025 [KY773196] | 0.014 | *Cren ; Thermoprotei* | *Thermogladius shockii* [NR_133017] | 83 |
