# Supplementary material for: Microbial Diversity in Sulfate-Reducing Marine Sediment Enrichment Cultures Associated with Anaerobic Biotransformation of Coastal Stockpiled Phosphogypsum (Sfax, Tunisia)
Source: Front Microbiol. 2017 Aug 21;8:1583. doi: 10.3389/fmicb.2017.01583 (PMC5566975; doi:10.3389/fmicb.2017.01583)
Supplement: Supplementary file 7 [file Table7.DOCX]

**Table S7. Phylogenetic affiliation of the OTUs affiliated to *Euryarchaeota* phylum obtained from the enrichment cultures of sulfate-reducing bacteria from marine sediment using sodium sulfate or phosphogypsum as sulfate source.**

Values in bold correspond to abundant OTUs (>1% of total sequences).

|  | Sequences per sample (%) | | | | | | | | | | | | Closest cultivated relative retrieved from NCBI nucleotide database | | |
| --- | --- | --- | --- | --- | --- | --- | --- | --- | --- | --- | --- | --- | --- | --- | --- |
| OTU no.  [GenBank number] | SA1 | SA2 | SL1 | SL2 | SF1 | SF2 | SPA1 | SPA2 | SPL1 | SPL2 | SPF1 | SPF2 | Taxonomy (phylum ; class ; order) | Species (Genbank number) | Identity (%) |
| 61437  [KY773191] | 0.000 | 0.000 | 0.007 | 0.079 | 0.000 | 0.000 | 0.000 | 0.004 | 0.000 | 0.000 | 0.001 | 0.000 | *Methanobacteria; Methanobacteriales* | *Methanothermobacter tenebrarum* NR_113002 | 82 |
| 240377  [KY773181] | 0.001 | 0.006 | 0.004 | 0.003 | 0.003 | **5.322** | 0.001 | 0.000 | 0.004 | 0.000 | 0.000 | 0.001 | *Methanococci; Methanococcales* | *Methanococcus voltae*  NR_104985 | 99 |
| 536415  [KY773182] | 0.000 | 0.000 | 0.000 | 0.000 | 0.001 | **1.062** | 0.000 | 0.003 | 0.001 | 0.000 | 0.000 | 0.006 | *Methanomicrobia; Methanomicrobiales* | *Methanocalculus pumilus* NR_028148 | 99 |
| 572253  [KY773184] | 0.001 | 0.006 | 0.007 | 0.029 | 0.000 | 0.167 | 0.000 | 0.000 | 0.000 | 0.000 | 0.277 | **0.587** | *Methanomicrobia; Methanomicrobiales* | *Methanomicrobium mobile* NR_044726 | 94 |
| 1141035  [KY773185] | 0.004 | 0.014 | 0.027 | 0.069 | 0.003 | 0.001 | 0.058 | 0.079 | 0.013 | 0.001 | 0.322 | **1.411** | *Methanomicrobia; Methanomicrobiales* | *Methanogenium marinum* NR_028225 | 98 |
| 851007  [KY773186] | 0.000 | 0.000 | 0.004 | 0.012 | 0.000 | 0.001 | 0.003 | 0.009 | 0.001 | 0.000 | 0.065 | **1.611** | *Methanomicrobia; Methanomicrobiales* | *Methanogenium marinum* NR_028225 | 98 |
| 187147  [KY773187] | 0.016 | 0.010 | 0.127 | 0.202 | 0.000 | 0.010 | 0.058 | 0.066 | 0.030 | 0.001 | 0.105 | 0.379 | *Methanomicrobia; Methanomicrobiales* | *Methanogenium marinum* NR_028225 | 99 |
| 238  [KY773188] | 0.004 | 0.017 | 0.065 | 0.143 | 0.000 | 0.001 | 0.036 | 0.045 | 0.012 | 0.009 | 0.622 | **3.691** | *Methanomicrobia; Methanomicrobiales* | *Methanogenium marinum* NR_028225 | 98 |
| 45108  [KY773189] | 0.013 | 0.010 | 0.019 | 0.029 | 0.000 | 0.014 | 0.004 | 0.009 | 0.003 | 0.009 | 0.006 | 0.017 | *Methanomicrobia; Methanomicrobiales* | *Methanogenium cariaci* NR_104730 | 94 |
| 46592  [KY773190] | 0.001 | 0.004 | 0.000 | 0.000 | 0.001 | 0.007 | 0.003 | 0.007 | 0.004 | 0.000 | 0.010 | 0.006 | *Methanomicrobia; Methanomicrobiales* | *Methanospirillum hungatei* NR_112982 | 99 |
| 87861  [KY773192] | 0.022 | 0.007 | 0.017 | 0.004 | 0.003 | 0.003 | 0.050 | 0.110 | 0.033 | 0.010 | 0.030 | 0.027 | *Methanomicrobia; Methanosarcinales* | *Methanosaeta harundinacea* NR_043203 | 96 |
| 561472  [KY773203] | 0.030 | 0.033 | 0.084 | 0.035 | 0.040 | 0.012 | 0.084 | 0.164 | 0.058 | 0.009 | 0.114 | 0.043 | *Methanomicrobia; Methanosarcinales* | *Methanosaeta harundinacea* NR_043203 | 98 |
| 535337  [KY773183] | 0.009 | 0.003 | 0.023 | 0.001 | 0.007 | 0.019 | 0.035 | 0.027 | 0.012 | 0.001 | 0.032 | 0.019 | *Methanomicrobia; Methanosarcinales* | *Methanosaeta harundinacea* NR_043203 | 99 |
| 826668  [KY773200] | 0.022 | 0.006 | 0.012 | 0.010 | 0.023 | 0.004 | 0.173 | 0.212 | 0.038 | 0.013 | 0.105 | 0.055 | *Methanomicrobia; Methanosarcinales* | *Methanosaeta concilii*  NR_102903 | 99 |
| Total OTUs (%) | 0.123 | 0.116 | 0.396 | 0.616 | 0.081 | 6.623 | 0.505 | 0.735 | 0.209 | 0.053 | 1.689 | 7.853 |  |  |  |
