# Supplementary material for: Microbial Diversity in Sulfate-Reducing Marine Sediment Enrichment Cultures Associated with Anaerobic Biotransformation of Coastal Stockpiled Phosphogypsum (Sfax, Tunisia)
Source: Front Microbiol. 2017 Aug 21;8:1583. doi: 10.3389/fmicb.2017.01583 (PMC5566975; doi:10.3389/fmicb.2017.01583)
Supplement: Supplementary file 8 [file Table8.DOCX]

# Table S8. Spearman’s rank correlation coefficients (r) between hydrogen sulfide production performances and microbial diversity indices, as well as relative abundances of phyla and proteobacterial classes in the enrichment cultures from marine sediments using phosphogypsum as sulfate source.

# Values in bold correspond to significant correlations (P < 0.05).

| **Variables** | **pH** | **Pmax (mM-H_2_S)** | **Vmax (mM-H_2_S/d)** |
| --- | --- | --- | --- |
| Simpson index | **-0.94** | **-0.83** | **-0.89** |
| Shannon index | **-0.83** | -0.66 | -0.77 |
| *Archaea* | 0.43 | 0.54 | 0.49 |
| *Actinobacteria* | -0.26 | -0.09 | -0.14 |
| *Bacteroidetes* | -0.03 | 0.09 | -0.09 |
| *Chloroflexi* | -0.49 | -0.31 | -0.37 |
| *Firmicutes* | **-0.87** | -0.71 | -0.66 |
| *Fusobacteria* | -0.54 | -0.26 | -0.60 |
| *Alphaproteobacteria* | -0.60 | -0.37 | -0.54 |
| *Betaproteobacteria* | -0.43 | -0.20 | -0.37 |
| *Deltaproteobacteria* | **0.94** | **0.83** | **0.89** |
| *Epsilonproteobacteria* | -0.77 | -0.60 | -0.71 |
| *Gammaproteobacteria* | -0.49 | -0.66 | -0.43 |
| *Spirochaetes* | **-0.89** | -0.71 | **-0.94** |
| *Synergistetes* | -0.14 | 0.09 | -0.20 |
| *Tenericutes* | -0.60 | -0.37 | -0.54 |
| *Thermotogae* | -0.31 | -0.54 | -0.14 |
